# Bubbles, Foam Formation, Stability and Consumer Perception of Carbonated Drinks: A Review of Current, New and Emerging Technologies for Rapid Assessment and Control

**DOI:** 10.3390/foods8120596

**Published:** 2019-11-20

**Authors:** Claudia Gonzalez Viejo, Damir D. Torrico, Frank R. Dunshea, Sigfredo Fuentes

**Affiliations:** 1 School of Agriculture and Food, Faculty of Veterinary and Agricultural Sciences, University of Melbourne, Melbourne, VIC 3010, Australia; Damir.Torrico@lincoln.ac.nz (D.D.T.); fdunshea@unimelb.edu.au (F.R.D.); sfuentes@unimelb.edu.au (S.F.); 2 Department of Wine, Food and Molecular Biosciences, Faculty of Agriculture and Life Sciences, Lincoln University, Lincoln 7647, New Zealand

**Keywords:** emerging technologies, quality control, foam-related parameters, artificial intelligence

## Abstract

Quality control, mainly focused on the assessment of bubble and foam-related parameters, is critical in carbonated beverages, due to their relationship with the chemical components as well as their influence on sensory characteristics such as aroma release, mouthfeel, and perception of tastes and aromas. Consumer assessment and acceptability of carbonated beverages are mainly based on carbonation, foam, and bubbles, as a flat carbonated beverage is usually perceived as low quality. This review focuses on three beverages: beer, sparkling water, and sparkling wine. It explains the characteristics of foam and bubble formation, and the traditional methods, as well as emerging technologies based on robotics and computer vision, to assess bubble and foam-related parameters. Furthermore, it explores the most common methods and the use of advanced techniques using an artificial intelligence approach to assess sensory descriptors both for descriptive analysis and consumers’ acceptability. Emerging technologies, based on the combination of robotics, computer vision, and machine learning as an approach to artificial intelligence, have been developed and applied for the assessment of beer and, to a lesser extent, sparkling wine. This, has the objective of assessing the final products quality using more reliable, accurate, affordable, and less time-consuming methods. However, despite carbonated water being an important product, due to its increasing consumption, more research needs to focus on exploring more efficient, repeatable, and accurate methods to assess carbonation and bubble size, distribution and dynamics.

## 1. Introduction

The assessment of bubble and foam-related parameters in carbonated beverages is of great importance since these are the main factors that determine the quality and acceptability of these beverages by consumers, as they have an influence on the perception of aromas, mouthfeel and flavor/taste. Carbonated beverages are one of the categories involved in the classification of the so-called aerated foods. These beverages include beer, carbonated water, carbonated soft drinks, and sparkling wine, among others. They can be classified according to the method used to carbonate the liquid, such as fermentation, natural carbonation, and gas injection, which are the three types of carbonation involved in the production of beverages [1,2]. Fermentation is the process by which yeast produces carbon dioxide (CO_2_) as a by-product and, therefore, CO_2_ becomes dissolved in the liquid, such as in the case of secondary fermentation during beer and sparkling wine production [3]. Natural carbonation occurs in products such as carbonated water, which is obtained from natural sources (springs) and do not require any further processing or treatment for CO_2_ production [4], whilst gas injection refers to the addition of CO_2_ in water, beer and other soft drinks at high pressure [2,5].

Within the fermented beverages drinks, beer is one of the most relevant, as it is the alcoholic beverage most consumed both worldwide and within Australia, specifically accounting for 78% and 66% in total volume sales in 2018, respectively, with a growth of 2.6% between 2016 and 2018 worldwide, and 3.1% between 2013 and 2018 in Australia [6]. In comparison, among the beverages produced by either natural carbonation or gas injection, the market for carbonated water, which is classified within the bottled water category, has been growing at a rapid rate in recent years as a healthier substitute for other soft drinks. It is also perceived as less “boring” than still water, due to the fizziness effect. Despite that, it only represents 10% worldwide [7,8] and 6.1% in Australia of the total volume sales within the bottled water category. Specifically in Australia, it represented a growth of 32% in the total volume of sales between 2010 and 2015, just 7% less than the still water growth [9]. Nowadays, consumers are becoming more demanding in terms of beverage quality and are looking for more premium products, especially regarding beer [10].

Therefore, the assessment and monitoring of final product quality factors are critical for the production of all carbonated beverages. In carbonated beverages, visual attributes linked to bubbles are directly related to their quality traits. This is due to the relationship between bubbles, and other sensory characteristics of the products, such as mouthfeel, release of aromas, and changes in tastes and flavors [2,11,12,13,14,15]. The main components in carbonated beverages that determine bubble characteristics, foam formation, and stability are the CO_2_ content and its source, as well as some tensioactive or surfactant substances such as proteins and sugars. All these components and compounds have a direct influence on beverage quality, hence the importance of their assessment and control [11,14,16,17,18]. Traditional methods to assess chemometrics, bubble and foam-related parameters of carbonated beverages tend to be time-consuming and involve high costs to small and medium companies, which make the process more inefficient, subjective and intuitive. Hence, it is important to develop modern techniques involving the use of new and emerging technologies, such as robotics, rapid non-invasive chemometric methods, such as near-infrared spectroscopy, and affordable electronic noses, and computer vision analysis to have more standardized measurements and reduce the human error factor and trial and error process, which currently dominates the industry.

## 2. Carbonated Beverages—Beer, Sparkling Wine, and Carbonated Water

Beer is the most ancient alcoholic fermented beverage, whose origins may trace back to 10,000 B.C. in the Neolithic era [19]. Its main ingredients are water, malted barley, yeast, and hops; however, other components may be used to increase its sensory properties and/or the amount of fermentable sugars, which are called adjuncts. The adjuncts usually consist of other sources of starch produced by milling such as maize, rice barley flour, or syrups, which help in the fermentation process as these are hydrolyzed during mashing into fermentable sources and dextrins [20]. In beer processing, a portion of the malted barley along with water and adjuncts are cooked for ~90 min, and temperature is progressively increased up to 100 °C. In this part of the process, the pH is adjusted to around 5.5–5.6, which is the optimum for enzyme activity [21,22]. Starch is gelatinized to make it more available for enzymes, and the system is sterilized. Once the mix is cooled down, it is mixed with the remaining malted grains and enters the mashing stage in which the non-fermentable starch is converted into low molecular weight fermentable sugars and proteins are converted into soluble proteins [20,22]. The following stage is lautering, whose goal is to separate the wort from the spent grain to clarify it. Then the wort is boiled along with hops to kill any microorganisms for 1.5 to 2 h, in which enzymes are also destroyed, proteins precipitate, and oxidation of the wort occurs; this makes the wort become darker in color [23]. After it is cooled, the following part of the process is pitching, in which yeast is added and pumped into the fermentation tanks. During fermentation, the yeast converts sugars into alcohol, and, during this process, one third of the carbon present in sugars is converted into CO_2_, nitrogen falls by around 33%, and pH drops to around 4.3 to make it safe for consumption. As the CO_2_ produced during fermentation is not enough for the desired foamability, beers may be either naturally or artificially carbonated; the first method is usually conducted in the bottle and consists of adding an extra amount of sucrose to allow the remaining yeast to produce additional CO_2_, while the artificial method is more frequently used and involves the injection of CO_2_ to achieve the desired carbonation [24]. The final stage in the brewing process is the packaging, in which the product is bottled against counter pressure of CO_2_ to avoid the loss of any gas and maintain the desired carbonation dissolved in the liquid [17,22].

Still wine, which is the base of sparkling wine, was first developed around 6000 B.C. in the Neolithic era when evidence of vessels with chemicals related to wine were found [25,26]. On the other hand, the history of sparkling wine is more recent and dates back to the 1660s in London and is linked to Christopher Merret, who published a paper in the Royal Society [27]. It consists of a low alcohol base wine, which undergoes a second fermentation to increase alcohol content and produce higher CO_2_. There are two main methods to produce this type of wine (i) Champagnoise or traditional, and (ii) Charmat [28]. The first method consists of an in-bottle second fermentation of the base wine, for which the addition of sucrose and yeast is required [29]. This fermentation is done at 12–20 °C for 15 to 45 days, followed by an aging period, which varies depending on the type of sparkling wine, but that usually takes >12 months. This aging period allows the wine to develop its characteristic aromas, flavors, complexity, and foamability. Following this period is the riddling, which consists of storing the bottles at 45° and turning them manually at progressively higher angles until they are virtually upside down, which ensures that yeast (lees) collects under the cap. Then, the disgorging takes place, in which the neck of the bottle is frozen and released with an ice plug and under the pressure contained in the wine bottle to remove the wine lees, and dosage is done using base wine with sugar to balance the acidity of the final product [30]. The Charmat method also uses base wine with low alcohol, but the second fermentation is conducted in stainless steel hermetically sealed tanks and with agitators to mix the yeast and added sucrose. In this method, the time of fermentation varies between 1 and 6 months, but the longer it is, the better the foamability and aroma retention. Once the fermentation is done, yeast is removed, and the wine is bottled at refrigeration temperatures under isobaric conditions. This is followed by aging with wine lees for at least 20 days [28,30].

Still bottled water was first produced in 1622 in the United Kingdom [31], followed by the earliest soft drinks, lemonade, and orangeade, which were developed in the 1660s [32]. On the other hand, the carbonated water production history traces back to the seventeenth century, in which the natural effervescence of water in spas became of interest. In 1741, Brownrigg was known to name the CO_2_ as mephitic air and started producing carbonated water from bicarbonate salts. In the late 1760s, Priestley discovered the way to produce artificially carbonated water using dissolved CO_2_ under pressure conditions. However, it was not until the 1770s that carbonated water in corked glass bottles started to be commercialized by Thomas Henry [33,34].

The main ingredients in carbonated water are mineral water and CO_2_, but there are some variations in which acidulants, additional minerals such as sodium bicarbonate, potassium sulfate, and sodium chloride, among others, and/or flavorings may be added. When CO_2_ is dissolved in water, it undergoes a reaction in which a hydrogen proton and bicarbonate ion are formed; this causes the pH of carbonated water to drop. Therefore, these types of water have a pH below neutrality, usually around 4 [35]. According to the Codex Standard for Natural Mineral Waters 108–1981 [36], there are different denominations for carbonated waters according to their carbonation source:

- Naturally carbonated natural mineral water: the gas in the water comes from the same source as the natural mineral water, and there is no loss or additional gas after packaging than the original content obtained from the source;
- Natural mineral water fortified with CO_2_ from the source: the bottled natural mineral water had a greater amount of gas than that obtained from the original source; however, the additional CO_2_ comes from the same source as the water;
- Carbonated natural mineral water: the bottled natural mineral water is carbonated by adding CO_2_ from a different source than the water.

There are also different subtypes of carbonated water according to their ingredients, the so-called sparkling water corresponds to either denomination: (i) naturally carbonated natural mineral water or (ii) natural mineral water fortified with CO_2_ from the source, depending on the manufacturer, and no additional ingredient is included. Soda water is the name given to the water containing sodium bicarbonate, and its pH may be regulated by adding an acidulant. Seltzer is the name commercially given to tap water, which is filtrated and artificially carbonated [37,38].

## 3. Bubbles and Foam of Carbonated Beverages

The term effervescence refers to the generation and growth of a large number of bubbles that rise through the liquid until they reach the surface, where they break up. In carbonated beverages, this cycle is repeated in a decreasing frequency within variable periods of time. This frequency is dependable on the growth time and the nucleation lapse time of a bubble [39]. A bubble consists of a small globule of gas separated from its liquid environment by either one of two interfaces. In carbonated beverages, the type of bubbles consists of one interface. A very important property of the interface is the surface tension, which is defined as the energy per unit area owing to the existence of the interface that is responsible for maintaining together the two halves of a bubble. Thus, the surface tension is responsible for the pressure differential between the internal and external parts of the bubble [2]. This is explained by the Laplace equation, which relates the internal and external pressures with the following equation (Equation (1)) [40]:

(1) Pb = P∞ +4 σ/d,

where *Pb* = Internal pressure of the bubble; *P*∞ = External pressure; *σ* = Surface tension and *d* = Diameter of bubble. The internal pressure is inversely proportional to the bubble size, hence the smaller the bubble, the higher the internal pressure. The main gas responsible for bubble formation in carbonated beverages is CO_2_, due to its high solubility in water, which tends to increase at a higher pressure and colder temperatures [2]. The solubility is explained by Henry’s law, which states that the concentration of dissolved CO_2_ in equilibrium (*c*) is proportional to the partial pressure of its gas phase (*P*). This is described by the following equation (Equation (2)) [41,42]:

(2) $$c\;=\;k_{h}P,$$

where *k_h_* = Henry’s law constant of gases. Bubbles can be formed by two different mechanisms: (i) homogeneous and (ii) heterogeneous nucleation. In carbonated beverages, heterogeneous nucleation is the mechanism responsible for bubble formation, as the homogeneous nucleation in these systems is thermodynamically forbidden due to the need of oversaturation above the 10^3^ that homogeneous nucleation requires, compared to the supersaturation of five times of CO_2_ concentration at 1 atmosphere pressure that carbonated beverages typically present [43,44]. In order to grow, bubbles need a catalytic site that typically consists of a gas pocket in a solid surface, which can be the glass wall and/or in the liquid phase. These gas pockets need a radius higher than the critical value, which is typically of 0.1–0.2 µm for carbonated beverages under standard temperature and pressure conditions. When the radius of gas pockets is lower than this critical value, the gas tends to dissolve, whereas, when its radius is equal to or higher than the critical one, it is able to grow into bubbles [7,45]. The bubbles’ radius is due to either the expansion or contraction of the gas inside the bubble or the flow of the gas; if this enters the bubble it will grow, whereas, when the gas leaves the bubble it will shrink. As previously detailed, an increase in gas flow in the bubble causes the rise of the internal pressure, which is mainly due to the surface tension, as explained by (Equation (1)). Furthermore, the bubble radius is mainly defined at the nucleation point, as described in the (Equation (3)) [11].

(3) $$B_{r}=\left(\frac{3R_{m}\gamma }{2\rho g}\right)$$

where *B_r_* = Bubble radius (m), *γ* = Surface tension (mN m^−1^), *ρ* = relative density of the liquid (kg m^−3^) and *g* = acceleration due to gravity (9.8 m s^−2^).

On the other hand, the rate of flow of gas to and from the bubble is explained by the mass transfer general equation (Equation (4)) [2,46]:

(4) Q = kA (C∞ − C*),

where *Q* = Molar rate of gas transfer to or from the bubble; *k* = Mas transfer coefficient; *A* = Surface area of the bubble; *C*∞ = Concentration of gas in the bulk liquid and C* = Concentration of gas in the liquid in equilibrium with the partial pressure of gas in the bubble. Thus, larger bubbles will grow faster due to their low internal pressure, low C*, and, consequently, a high molar rate of gas transfer (*Q*) [2]. Food and beverages, which have been aerated, tend to be thermodynamically unstable, and the stability of bubbles is primarily given by the rheological properties of the product, which are dependent on the surfactant substances present [47]. Therefore, carbonated water is naturally the less stable carbonated beverage due to its lack of viscosity and tensioactive or surfactant substances, which prevent foam formation and lead bubbles to break when reaching the surface of the liquid. In contrast, beverages such as sparkling wine and, to a greater extent, beer, whose composition consists of proteins and carbohydrates, have a higher viscosity than water, but low enough to allow bubbles to ascend. The growth rate and the rising velocity are also dependent on the availability of CO_2_ concentration in the liquid and the presence of tensioactive substances such as proteins and sugars [11,48,49]. The tendency of larger bubbles to coalesce and the bubble velocity are described by the Stoke equation (Equation (5)) [2,46]:

(5) $$v_{st}\;=\;g\rho ld^{2}/18µ,$$

where *v_st_* = Ascending velocity of one bubble; *g* = Acceleration due to gravity; *ρ_l_* = Density of liquid; *d* = Diameter of bubble and µ = Viscosity of liquid.

In beverages such as beer and sparkling wine, the bubbles form when opening the bottle, which allows freeing the gas dissolved in the fluid, followed by their rise through the liquid, and surfactant or tensioactive substances, which are able to increase the viscosity of the bulk phase and decrease the drainage velocity of the lamellae’s (thin film in the liquid—gas interphase) fluid, and, therefore, allow the bubbles to rest in the surface of the liquid, forming a layer of foam [14,17,49,50]. The foam formation in the glass also depends on the pouring method, temperature of the liquid, and CO_2_ concentration in the beverage [51,52]. Lower temperatures are preferred, especially for beer and sparkling wine, because, as previously mentioned, CO_2_ solubility increases [2] and, therefore, avoids an excessive foam formation, which is often desired by consumers [13]. The role of proteins in foam stability is due to their structure, which presents molecules of both hydrophilic and hydrophobic properties. Once the protein chain unfolds at the bubble’s interphase, the hydrophilic molecules remain in the liquid, while the hydrophobic molecules are in contact with the air. This allows the proteins to form a layer at the interface, which provides foam stability [2,49,53]. Furthermore, foam texture, especially in beer, depends on the bubble size distribution; thus, when higher amount of small bubbles are present and the liquid fraction in the foam is greater, it leads to foam creaminess, which is often preferred by consumers [11]. In contrast, when the bubble size distribution is higher, it leads to a coarse foam [49,54].

Bubbles can lead to different states of less stability, such as disproportionation or coalescence, mainly caused by the bubble size distribution. Disproportionation is usually due to wide bubble size distribution, which leads the smaller bubbles that present high Laplace pressure to disperse into the larger bubbles with lower pressure, hence it provokes them to break more readily and to reduce foam stability; this is explained by the De Vries equation (Equation (6)) [11]:

(6) $$r_{t}^{2}=r_{0}^{2}-\frac{4RTDS\gamma t}{P\theta },$$

where *r_t_* = Bubble radius at time t, *r*_0_ = Bubble radius at time 0, *R* = Gas constant (8.3 J K^−1^ mol^−1^), *T* = Absolute temperature (K), *D* = Gas diffusion coefficient (m^2^ s^−1^), *s* = Solubility of the gas (mol m^−3^ Pa^−1^), *γ* = Surface tension, *t* = Time (s), *P* = Pressure and *θ* = Film thickness between bubbles.

On the other hand, coalescence occurs when the lamellae are broken, leading two small bubbles to join and form a larger one and, therefore, decreasing its internal pressure. Another factor that determines foam stability in beer and sparkling wine is the foam drainage, which occurs due to the weakening of the foam layer provoked by gravity and the Plateau border (intersection between three bubble films) suction, which cause the bubbles to collapse, therefore, if foam drainage occurs at a higher rate, the foam will be less stable; this is explained by the (Equation (7)) [11,45]:

(7) $$Q=\frac{2\rho gq\delta }{3\eta },$$

where *Q* = Flow rate (m^3^ s^−1^), *η* = Viscosity of the film liquid (Pa s), *ρ* = Relative density of the beer, *q* = Length of the Plateau border (m), *g* = Acceleration due to gravity and *δ* = Thickness of the film (lamella; m).

These concepts have been applied by researchers to assess bubble and foam-related parameters in the different carbonated beverages using either the traditional methods or more novel methods using emerging technologies such as computer vision and robotics.

## 4. Methods to Assess Bubble and Foam-Related Parameters

Quality, foam behavior, and gas-phase parameters are the three main methods to assess air or gas incorporation in food and beverages: (i) food quality involves parameters related to appearance; (ii) rheology and/or (iii) texture of the product. The most representative parameters of the foam behavior category are the foamability (capacity of foam formation) and foam stability. Gas-phase parameters refer to the assessment of bubble size distribution, individual bubble behavior and gas content [2].

Several methods to assess gas phase parameters and foam behavior in carbonated water, beer, and sparkling wine have been developed. While there are not many recent studies of bubbles in carbonated water, Barker et al. [55] used image analysis to measure bubble nucleation and growth using the Image-Pro Plus software (Datacell, Maidenhead, UK), which was able to convert the images into binary data, and obtained the average diameter of bubbles. Kappes et al. [56] measured the carbonation level in carbonated beverages, including carbonated water, using the Zahm and Nagel puncturing device (Zahm & Nagel Co., Inc., Holland, NY, USA). Moritaka et al. [57], produced different samples of carbonated drinks using corn syrup, sodium citrate, and citric acid plus distilled water and recorded images using ImageJ software (Wayne Rasband, Public domain) and they were processed five times by evaluators, who measured the number of bubbles, average and total area of the bubbles. Liger-Belair et al. [7] measured bubble dynamics in waters with different levels of carbonation. In their study, they assessed the lifetime of clouds of bubbles by manually pouring the water in a flute type glass using a cold light as background at room temperature and quantified time manually using a chronometer. Furthermore, the authors measured the loss of dissolved CO_2_ in water by placing the flute on a scale during the pouring and monitoring the weight difference. On the other hand, they measured the bubble growing kinetics using a plastic goblet and a cold backlight by taking high-speed images using a macro objective and monitoring the bubbles growth 5 min after pouring and during 30 s. Those three measurements were done independently. More recently, Gonzalez Viejo et al. [58] developed a method to assess bubble size and bubble size distribution of carbonated water by capturing images of the samples in a Petri dish and analyzing them using a customized code written in Matlab^®^ (Mathworks Inc., Natick, MA, USA) based on computer vision analysis. This algorithm is able to identify every single bubble and measure their diameter in pixels to classify them in small, medium and large.

In beer, there are several methods used to assess foamability, foam stability, and/or foam drainage. Table 1 shows the different traditional methods and their techniques, as well as some of the most recently developed techniques [11,14]. Those methods can be categorized mainly into two groups according to their foam formation: (i) artificially using CO_2_ pressure and (ii) naturally through manual or automatic pouring. The methods belonging to category (i) are the National Institute for Malting Barley, Malt and Beer (NIBEM) (NIBEM-T; Haffmans BV, Venlo, Holland) [59,60], foam flashing [59], Rudin [61], Steinfurth foam stability (Steinfurth, Inc., Marietta, GA, USA) [62], shake test [63], Carlsberg automated analysis [64], Blom [51], foam-lacing [65] and the low-cost image analysis system [66]. These methods induce foam formation by applying pressure using CO_2_ [11,66]. However, since this is not the natural foam formation process, these methods measure the capacity of foamability and/or foam stability of the beers, but not the real performance of each bottle. Therefore, these may be used to assess the quality of raw material and beer formulation but not to assess the quality of individual beer bottles and their sealability. Furthermore, all, except for the shake test and foam-lacing test, measure the beer samples between 20 and 25 °C, which are not the usual consumption temperatures of 4–14 °C according to the beer style [66,67]. On the other hand, the methods belonging to category (ii) are the sigma value [59], Constant method [68], foam cylinder method [69], Ross and Clark [70], foam collapse time and RoboBEER (University of Melbourne, Melbourne, VIC, Australia) [14,71,72]. These methods assess foamability and foam stability by natural formation through either manual or automatic pouring, which simulate the real way of consumption, except for the Ross and Clark method, which alters the beers by degassing the samples [11]. In contrast with methods from category (i), which use CO_2_ pressure, most of those from category (ii) measure beer samples at refrigeration temperatures (4–6 °C), which are the regular consumption temperatures for most beers.

A drawback of most methods from both categories, except for the NIBEM, Steinfurth, Carlsberg, the low-cost image analysis system, and RoboBEER, is that the analyses are conducted manually and visually, which may lead to human errors. Furthermore, all methods except for the Constant Pour test and RoboBEER only measure one or two parameters (Table 1), which consist of half-life of foam, foam collapse time, foam stability, or lacing in the case of foam-lacing method. Of all the methods, RoboBEER, which is an automated technique that consists of a robotic pourer constructed with LEGO^®^ (The Lego Group, Billund, Denmark) blocks and servo motors along with low-cost sensors controlled by Arduino^®^ boards (Arduino Computing platform, Ivrea, Italy), measures the highest number of parameters (15 parameters), related to foam, bubbles and color as well as CO_2_ and alcohol. This method is also able to assess the sealability of the final product, as reported by Gonzalez Viejo et al. [14]. RoboBEER is coupled with computer vision analysis using a systematic code developed in Matlab^®^; the algorithm can identify and measure the volume of the liquid and foam within time. This is then able to calculate parameters such as maximum volume of foam, and foam stability in two ways: (i) calculating the time that foam lasts from time 0 up to 5 min (total lifetime of foam) and (ii) calculating the area under the curve from the maximum volume of foam until the 5 min, which is the duration of the videos. A limitation of this method to highlight is that it is required to adjust the number of rotations and delay times according to the size and weight of the bottle being measured; however, this may be fixed by adding a sensor to detect the bottle dimensions and weight and automatically adjust the settings [14].

Foam measuring methods were first developed for beer around the 1930s [78]; however, due to the increasing interest in assessing foam in carbonated beverages due to its relationship with the products’ quality, some beer methods, such as Ross and Clark and Rudin, have been tested in sparkling wine [70,79]. More specific methods for sparkling wine foam assessment have been developed, such as the most widely used Mosalux method [80], which consists of an adaptation of the Rudin method measuring the interruption of a beam of ultra-red light using an infrared emitter and receiver, and is able to measure three parameters (i) maximum foam height, (ii) foam stability height and (iii) foam stability in time [81]. Another method named Computerized Assisted Viewing Equipment (CAVE) is an automated technique, which consists of a robotic pourer assisted by a computer and connected to a data recording system with three video cameras at different angles; this system is able to assess maximum foam thickness, total time of pouring, minimum and maximum height of foam, and velocity of foam and liquid [82]. Other techniques, such as FIZZeyeRobot (University of Melbourne, Melbourne, Vic, Australia) and the free pour method, involve the automatic foam assessment using computer vision algorithms. The FIZZeyeRobot consists of an automatic robotic pourer, from which 1–2 min videos are taken to be further analyzed using computer vision with similar algorithms to the RoboBEER method for beer, and is able to measure parameters such as average lifetime of foam, initial height, height, velocity and time of collar, drainage, foam expansion, foam velocity, volume of foam, maximum volume time, percentage of wine in the foam, and ratio of small bubbles in the foam [83]. The free pour method consists of a manual pourer with two cameras (top and side); the videos are analyzed using computer vision analysis with the ImageJ software, and it is able to obtain parameters such as maximum and minimum height of foam, foam stability and width of collar [84]. On the other hand, Liger-Belair et al. [85] assessed bubble dynamics in sparkling wine from enlarged images taken from one side of the glass using a stroboscope and a flashlight on other sides of the glass.

## 5. Carbonated Beverages Quality Based on Sensory Analysis

### 5.1. Descriptive Sensory Analysis

Descriptive sensory analysis is traditionally conducted using a trained panel and has the purpose of developing the sensory profile of a product by evaluating the intensities of the main descriptors. This type of sensory analysis is usually used in new product development and to assess the quality of the same formulation in the different batches produced. The traditional and most common descriptive method is the quantitative descriptive analysis (QDA^®^), which consists of a 15 cm non-structured scale with 10–16 trained panelists [86,87], although some authors use a shorter continuous scale of 10 cm or 10 points. In the case of carbonated beverages, this type of sensory test is usually used as another method to assess the intensity of foamability, foam stability, bubbles, and/or carbonation [11,14]. While there are not many studies using descriptive sensory in carbonated water, carbonation mouthfeel is among the most explored attributes. This consists of the sensation which irritates the trigeminal nerve due to the carbonic anhydrase that is released when bubbles burst, and that provides different descriptors such as tingling, burning, prickling, and numbing, among others [88]. Harper and McDaniel [89] evaluated the effects of temperature and CO_2_ in the sensory rating of different descriptors such as bubbly, size of bubbles, sound of bubbles, gas expansion, burn, numbing, and bite mouthfeel to mention a few, using a scale from 0 = none to 15 = extreme. The authors found that cooling, bite, burn and numbing mouthfeel were higher are cooler temperatures (3 °C), while bubble size and sound increased at higher temperatures (10 °C). Rey-Salgueiro et al. [90] used a scale from 0 = absence to 10 = intense to assess different descriptors related to taste, texture, and appearance to relate them to the chemical components of different carbonated waters. Likewise, Dessirier et al. [91] used a 10-point scale from 0 = absent to 10 = very strong to assess the carbonation sensation in the tongue. Kappes et al. [56] used a 15 cm scale to rate different sensory descriptors in flavored carbonated beverages and found a positive correlation between the level of carbonation and sourness (*r* = 0.79), and a negative correlation with astringent (*r* = −0.82) and bitterness (*r* = −0.88).

In beer, there have been more studies using descriptive analysis to assess attributes related to foam and carbonation. Da Costa Jardim et al. [92] assessed Brazilian beers using QDA^®^ analysis with a panel of eight subjects to evaluate 15 descriptors related to flavor, taste, and appearance, such as foam persistence. Similarly, Medoro et al. [93] evaluated Italian beers using QDA^®^ to assess 28 descriptors of aromas, taste, appearance including foam persistence, and mouthfeel, which included carbonation. However, although these two studies claim that they conducted a QDA^®^ method, they used a nine-point scale, which is not the traditional 15 cm or 6-inch scale used for this technique [86,87].

Descriptive sensory analysis has also been used to assess differences in foam-related parameters of different beer styles or treatments. Bobková et al. [94] used the International Standard ISO 8586-2:2008 [95] to train the panelists and assess beers with saccharose using a nine-point scale and found that foam stability decreased with the addition of saccharose. On the other hand, Gonzalez Viejo et al. [14] assessed different beer samples from the three different types of fermentation using QDA^®^ with a 15 cm non-structured scale and found that the spontaneous fermentation beers had the highest foamability and foam stability. Furthermore, these authors found a positive relationship between the level of CO_2_ and the intensity of sour taste, as well as some aromas, such as floral, spicy and burnt sugar. Recently, more novel techniques have been developed using robotics and computer vision (RoboBEER method), and machine learning, specifically artificial neural networks (ANN) to assess ten different sensory descriptors, such as bitter, sweet and sour tastes, grains, hops and yeast aromas, viscosity, astringency, carbonation mouthfeel and hops flavor using the 15 color and foam-related parameters obtained from RoboBEER (Table 1), with a very high accuracy with correlation coefficient *r* = 0.91. This was possible due to the influence of color and foam-related parameters of the aforementioned sensory descriptors. Furthermore, this method was tested in beer samples brewed using audible sound during fermentation and carbonation with high accuracy (R = 85) [96]. Therefore, this technique aids in the fast-screening of beer samples to reduce time and costs that the traditional sensory sessions involve, with the advantage that this method is able to obtain both physical and sensory parameters and it may be used for any other carbonated beverage by using the corresponding targets for ANN models.

In sparkling wine, apart from the descriptive analysis, studies using tests such as temporal check all that apply (TCATA) have been used. McMahon et al. [97] assessed some descriptors related to carbonation, such as bubble pain (pain felt in mouth when bubbles burst), creaminess (smoothness given by small or dense bubbles) and foamy (sensation of foam expanding in mouth) mouthfeel, with a trained panel using a 15-cm continuous scale with anchors at 1.5 = low and 13.5 = high in sparkling wines with different sugar types and levels. McMahon et al. [98] compared two sensory methods, descriptive analysis with a 15 cm scale and TCATA, to assess sparkling wines with different carbonation levels; in this study, the mouthfeel of different carbonation-related descriptors, such as burning, numbing, bubble pain and foamy, among others, were evaluated. On the other hand, White and Heymann [99] evaluated the sensory profile of sparkling wines over time and described that they used a “generic” sensory descriptive test with a trained panel; however, they did not specify which method and scale they used. Culbert et al. [100] found a separation of sparkling wines into their production method based on their sensory profile for attributes such as aromas (floral, toasty, confectionery and tropical), and other attributes as sweetness and meaty/savory flavor, among others, obtained using a 15 cm scale.

### 5.2. Consumer Sensory Analysis

**Figure 1:**
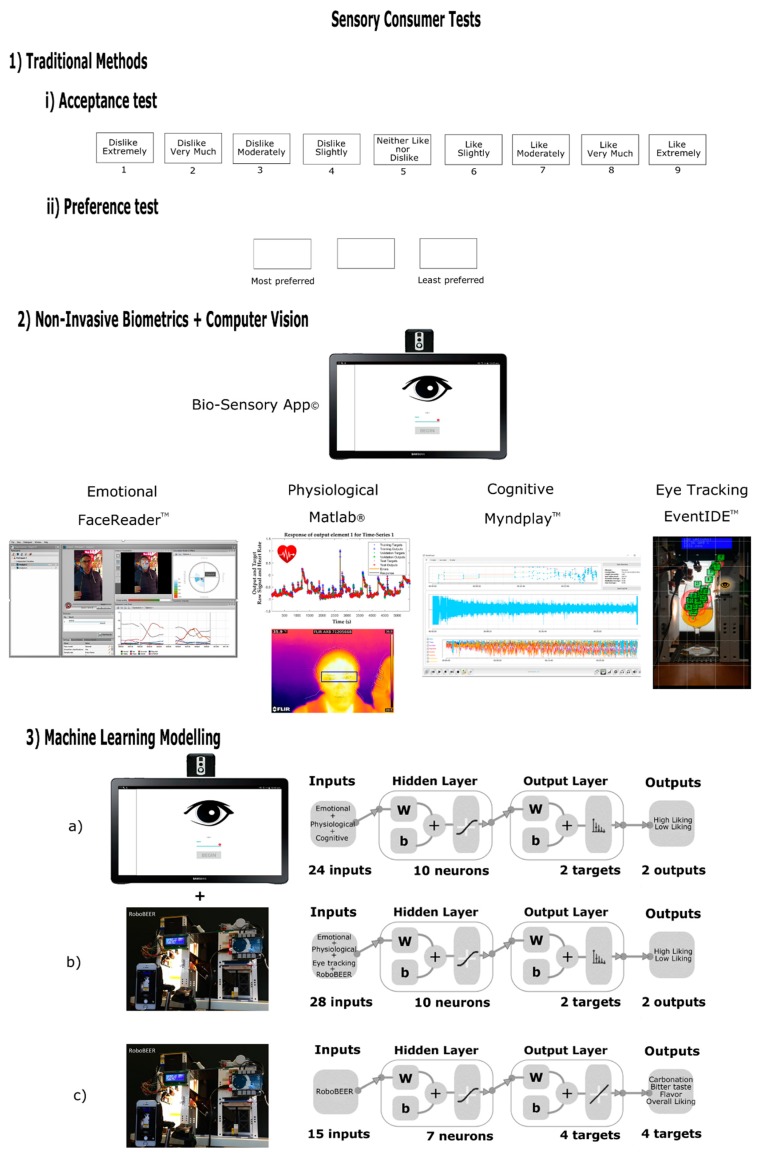
Representation of sensory acceptability methods to assess carbonated beverages, including the traditional and more advanced techniques including non-invasive biometrics, robotics, and machine learning techniques. Showing the model diagrams to assess (**a**) low and high levels of liking of carbonation mouthfeel, flavor and overall liking using biometrics as inputs, (**b**) low and high levels of liking of foam using biometrics and color and foam-related parameters as inputs, and (**c**) rating of carbonation mouthfeel, bitter taste, flavor and overall liking using color and foam-related parameters as inputs.

Consumer sensory tests are highly relevant to assess the most important attributes of a product that are related to quality perception and acceptability. The most popular consumer sensory methods are the traditional 9-point hedonic scale (1 = dislike extremely, 5 = neither like nor dislike, 9 = like extremely) and preference test based on either ranking (Figure 1) or choice of the preferred sample [87,101]. Similar to descriptive sensory analysis, there are not many studies using acceptance sensory tests in carbonated water. However, in the few published studies, authors have used different scales or tests to assess liking or preference. Risso et al. [102] used a 150 mm continuous scale from “not at all” to “very much” with 28 consumers, which is not a typical or recommended scale to be used with consumers. In that study, the authors assessed the perception and expectations of waters with different carbonation levels contained in glasses with different colors, finding that red glasses elicit a perception and expectation of higher carbonation. Barker et al. [55] used a paired comparison test to assess whether the participants were able to identify the sample with highest carbonation using samples with different residual CO_2_ and with small and medium bubbles, concluding that most consumers prefer smaller bubbles; however, this study must be conducted with higher number of consumers, as only 17 participants were used, which is not enough to find significant differences. On the other hand, Zampini and Spence [103] evaluated the carbonation of waters from the effect of sound with 24 consumers using a scale from 0 = still to 100 = sparkling, concluding that the perceived carbonation level and oral irritation were not influenced by different carbonation sounds. According to Des Gachons et al. [104], the carbonation mouthfeel increases thirst-quenching when compared to still beverages; therefore, some authors [55,105] have attempted to alter bubble size in sparkling water by injecting CO_2_ to modify the fizzing sensation. More recently, Gonzalez Viejo et al. [58] applied audible sound (25–75 Hz) to modify bubble size in commercial carbonated water and found that it increased consumers’ acceptability (nine-point hedonic scale). A similar treatment was applied to reduce bubble size and increase foamability in beer, which may be used to increase quality [96]. This method may be tested for its effect on thirst-quenching to find if the bubbles’ modification has any effect on it.

There have been some studies in beer to assess consumer acceptability, including their perception based on foam and carbonation. Hong et al. [106] used a 9-point hedonic scale to assess the acceptability of beers among Korean consumers, evaluating attributes including foam volume, total CO_2,_ and density, among others. Donadini et al. [107] assessed different sensory descriptors, such as carbonation, body, and alcohol, among others, using a hedonic scale with consumers from three different countries: Italy, Poland, and Spain. Other authors have assessed beer acceptability based on the visual assessment of foam using images of three different beers: (i) flat, (ii) medium foam and (iii) high foam, using different techniques, such as the path analysis method of eliminating preferred stimuli [108], preference test with ties and “none” option [109] and using a 7-point Likert scale [110]. In general, these studies concluded that consumers prefer beers with moderate or medium level of foam.

Recently, another method involving the use of non-invasive biometrics has been used to assess consumers acceptability from conscious and subconscious (emotional, physiological) responses (Figure 1). This method consists of an automated integrated camera system, which includes an infrared thermal FLIR AX8™ camera and Android^®^ tablet coupled with a Bio-Sensory application to display the sensory questionnaire; the method also involves the analysis of videos from participants using computer vision to assess eight emotions, two dimensions (valence and arousal), heart rate and body temperature [111]. Gonzalez Viejo et al. [13] used this method along with an electroencephalogram (EEG) device to assess brain wave responses to assess beers from different types of fermentation and foamability and used ANN to develop three models to classify beer samples into low and high levels of liking of (i) flavor, (ii) carbonation mouthfeel and (iii) overall liking using only the subconscious responses as inputs; all models presented high accuracy >80% (Figure 1a). Furthermore, Gonzalez Viejo et al. [112] evaluated the perceived quality and liking of foam-related parameters from visual assessment using videos from the pouring of beer samples from the RoboBEER to uniform the pouring and using the integrated camera system and Bio-Sensory App along with eye-tracking; the authors were able to develop an ANN model to classify beers into low and high level of liking of foamability with 82% accuracy using only the biometric responses from consumers as inputs (Figure 1b). On the other hand, a more cost-efficient and less time-consuming instrumental method was developed using robotics and machine learning by measuring the 15 color and foam-related parameters with the RoboBEER method as inputs for an ANN multi-target regression model to predict the mouthfeel carbonation, bitter taste, flavor and overall liking, in which the outputs are given in a 9-point hedonic scale (Figure 1c) [113]. These novel methods may be applied to any other carbonated beverage and machine learning models may be developed using the corresponding targets for the specific products.

In sparkling wine, there have been more studies using descriptive sensory methods than consumer tests. However, authors such as Culbert et al. [114] conducted a study to assess consumer acceptability of Australian sparkling wines from different production methods. In that study, the authors used a 9 cm hedonic continuous scale and found that wine produced with the Charmat method was the most liked. On the other hand, McMahon et al. [97] assessed the consumer acceptability of sparkling wines with different types and levels of sugar using a nine-point hedonic scale and evaluated the liking of different sensory attributes, such as carbonation mouthfeel, foamy and overall acceptance, among others, finding a strong and positive correlation between overall liking and liking of carbonation (*r*^2^ = 0.95) and foamy liking (*r*^2^ = 0.94).

As Figure 1 shows in the summary of the available methods to assess sensory acceptability that may be applied to any carbonated beverages, the more advanced methods involve the use of machine learning modeling, biometrics, and/or robotics. Machine learning is an emerging technology that has been recently applied as a potential fast-screening tool to assess acceptability of multiple new beer products, which aids in the elimination of the fatigue limitation of consumers due to a large number of prototypes in the products development stage. Furthermore, specifically ANN has as an advantage that predictive models may be developed using multitargets, which also reduces the time of analysis. This offers more reliable, more affordable and less time-consuming techniques to assess consumer perception and acceptability for carbonated beverages, that may be focused on their color and foam-related parameters.

## 6. Future Trends

Due to the increasing interest and importance of the development and application of new and emerging technologies in food control and, more specifically, in the quality assessment of carbonated beverages based on the foamability, foam stability and bubbles, trends are more focused in the development of automated, cost-effective and less time-consuming physicochemical and sensory methods using robotics, computer vision and machine learning. However, even though these have been successful in the assessment of beer color and foam-related parameters as well as the sensory descriptors and consumer acceptability, they have not yet been developed for other beverages, such as carbonated water and sparkling wine. Despite that there are already methods involving computer vision and robotics for sparkling wine, the application of machine learning and biometrics to predict sensory attributes have not been explored yet. While carbonated water is an important product due to the increasing consumption, there are not many studies exploring efficient methods to assess the carbonation, and bubble size, distribution, and dynamics. The application of the emerging technologies for quality assessment of carbonated beverages based on bubble and foam-related parameters would potentially allow their implementation in the industry to evaluate the products within the production line in real-time. This would aid in the quality control to detect defects in these parameters on time and avoid any economic losses due to low-quality products in the market.

## Figures and Tables

**Table 1:** Methods to assess foam-related parameters in beer and their working conditions.

Method	Foam Formation	Parameters	Time	Technique	Sample Temperature (°C)	Reference
NIBEM	CO_2_ Pressure	Foam stability Foam temperature	Varies depending on sample	Automatic	20 °C	[59,60]
Sigma value	Manual pouring	Foam collapse rate	~5 min	Manual—Visual	22–27 °C	[59]
Foam flashing	CO_2_ Pressure	Foam collapse rate	100 s	Manual—Visual	25 °C	[59]
Constant method	Manual pouring	Foam height Head Foam stability Half-life of foam Normalized half-life Density of foam Quality of foam	20–25 min	Manual—Visual	4 °C	[68]
Foam cylinder method	Manual pouring	Volume of foam Foam collapse rate	15 min	Manual—Visual	4 °C	[69]
Rudin	CO_2_ Pressure	Foam stability	~10 min	Manual—Visual	20 °C	[61]
Ross and Clark	Manual pouring	Foaminess (time)	5 min	Manual—Visual	15 °C	[70,73]
Steinfurth foam stability	CO_2_ Pressure	Foam stability Foam decay	Varies depending on sample	Automatic	20 °C	[62,74]
Shake test	CO_2_ Pressure—Shaking	Foam stability	30 min	Manual—Visual	4 °C	[63,75]
Carlsberg automated analysis	CO_2_ Pressure	Half-life of foam	~8 min	Automatic	15–25 °C	[64]
Foam collapse time	Automatic pouring	Foam collapse time	Varies depending on sample	Computer vision Manual/Semi-automatic	6 °C	[76]
Blom	CO_2_ Pressure	Foam stability Half-life of foam	≥5 min	Manual—Visual	20 °C	[51]
Foam–lacing	CO_2_ Pressure	Lacing	~15 min	Manual—Spectrophotometer	10 °C	[65,75,77]
Low-cost image analysis system	CO_2_ Pressure	Half-life of foam Beer–foam interface height	Varies depending on sample	Automatic—Computer vision	20 °C	[66]
RoboBEER	Automatic pouring	MaxVol TLTF LTF FDrain SmBubb MedBubb LgBubb Color: RGB CIELab Alcohol CO_2_	5 min	Automatic—Computer vision	4 °C	[14]

Abbreviations: MaxVol = maximum volume of foam; TLTF = total lifetime of foam; LTF = lifetime of foam; FDrain = foam drainage; SmBubb = small bubbles; MedBubb = medium bubbles; LgBubb = large bubbles; RGB = red, green, blue; CO_2_ = carbon dioxide; NIBEM = National Institute for Malting Barley, Malt and Beer.
